# Width identification of transition zone between desert and oasis based on NDVI and TCI

**DOI:** 10.1038/s41598-020-65286-5

**Published:** 2020-05-26

**Authors:** Shuxin JI, Xuelian Bai, Rongrong Qiao, Lixiang Wang, Xueli Chang

**Affiliations:** Department of Resources and Environmental Engineering, Ludong University, Yantai, 264025 P.R. China

**Keywords:** Ecology, Environmental sciences

## Abstract

The oasis-desert transition zone, the boundary between the desert and oasis, has special significance in maintaining oasis stability and indicating ecosystem health. The width of the boundary is one of the critical indicators to determine the sampling design and restrict findings scaling in the study of the desert oasis transition zone. Buffer analyze and focal analyze were conducted to determine the width among oasis-desert transition zone and oasis artificial sand fixation zone in Hexi corridor China. Focal analyses indicate that TCImax and TCImin can constrain NDVI of trend variation, and the effect increases with the analysis scale. On the same spatial scale, NDVI and TCI have opposite trends and have intersections. The intersection of the sandy desert transition zone is between 30–90 m, and the oasis artificial sand-fixaion zone is between 90–150 m. The width of the sandy desert transition zone is between 220–300 m, and the width increases with the increase of analysis scale. The oasis artificial sand-fixation zone is between 420 and 540 m, which decreases with the increase of the analysis scale. NDVI shows a trend of decreasing from the oasis boundary to the desert, the trend of TCI is different from that of NDVI, showing an increase from the edge of oasis to the interior of desert. The differences in the spatial distribution of NDVI and TCI can be clearly expressed, and different types of transition zones and analysis scales have their own characteristics.

## Introduction

In the study of ecology and environments sciences in arid zones, the oasis-desert transition zone is the basic unit of much ecological hotspot research, and it has a particular indicative function in the study of oasis ecosystem stability and regional biodiversity conservation^1,2^. To determine the inside and outside boundary of the oasis-desert transition zone is the key to determining the width of the transition zone because the width of the zone will affect the sampling location selection, sample layout scheme, and research result scaling directly. The transformation of material and energy in the transition zone between different ecosystems is drastic and frequent, and the local environment in the transition zone is more sensitive to external natural disturbances such as precipitation and fire, which is an “indicator” of natural environmental changes. at the same time, this area also provides natural shelter for many insects, birds and small mammals. Therefore, the identification of transition zone width between different systems plays an important role in local biodiversity conservation and monitoring regional environmental changes. From the perspective of the spatial attributes of the oasis-desert transition zone, the inner boundary of the farmland and woodland is easy to identify, but the outer boundary is different in various scales and regional studies^3,4^. This difference comes from the spatial heterogeneity of vegetation types and the diversity of oasis boundary pattern. It can be inferred that the outer boundary of the oasis-desert transition zone is a variable whose change is related to vegetation and micro-meteorological conditions and the spatial position changes as the boundary bend. Thus, it is an effective way to identify the boundary width of the oasis-desert transition zone employing the characteristics of vegetation and micro-meteorology.

## Application of Vegetation Index

Several studies have documented the Vegetation Condition Index (VCI), the Temperature Condition Index (TCI), and the Water Supplying Vegetation Index (WSVI) were used based on various remote sensing data. Consistent findings have been obtained to identify crop drought conditions^5,6^. The core conclusion of these findings was that VCI and TCI could be used to calculate surface vegetation conditions and temperature^5,7^, and VCI and TCI interactions were used to construct a Vegetation Temperature Condition Index (VTCI)^8^. Since the various indexes mentioned above can capture the gradient difference between the productivity levels of the vegetation ecosystem, it is also possible to obtain the surface drought condition (inversion by surface brightness) using the thermal infrared band. Then, it is methodologically feasible to directly use the Normalized difference vegetation index (NDVI) and TCI to detect the gradient of vegetation and surface temperature outside the oasis boundary. As a result of the surface vegetation conditions and brightness, temperature changes are different^9–11^, and the normalization index can be used to determine the intersection point, and the trend changes inflection point on the spatial gradient.

The research had two main objectives. The first one is to detect the width of the oasis-desert transition zone, which is determined by the intersection and inflection point between NDVI and TCI. The second objective was to compare different ecological systems and observe their different width changing trends depending on varies transition zone.

## Study area and Methodology

### Study area

**Figure 1:**
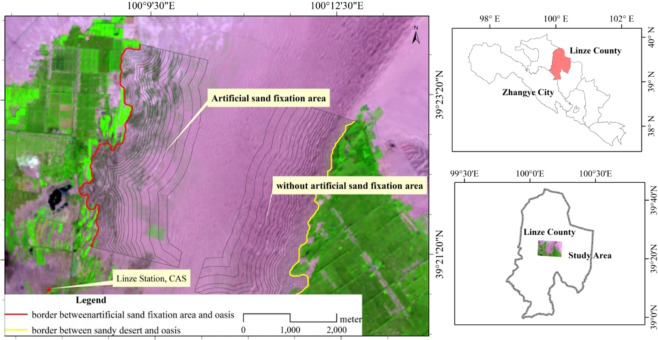
Location of the study area and oasis borders (red and yellow lines).

The study area is located at the Hexi Corridor Heihe River, near the oasis-desert transition zone of the Linze Inland River Basin Comprehensive Research Station of the Chinese Academy of Sciences (Fig. 1). The marginal expansion of this area is representative in China’s oasis area^12^. The annual average precipitation in the study area is 117 mm, of which 65% is from July to September; the annual average temperature is 7.6 °C, and the average evaporation is 2390 mm. There are 4 boundary types in the study area, namely oasis and sandy desert, artificial sand-fixation area, gravel desert, and stony bare mountain. Because this study is the first to use the NDVI and TCI trends to determine the width of the oasis-desert transition zone, the natural boundary between the oasis and sandy desert boundary types (no artificial sand-fixation, yellow line in Fig. 1) and artificial interference boundaries are selected. (Manual sand-fixation area, the red line in Fig. 1) Two types were analyzed. The main plants in the study area are arbors such as *Populus gansuensis* C. Wang et H. L. Yang, *Elaeagnus angustifolia* Linn., shrubs like *Caragana Korshinskii* Kom, *Hedysarum Scoparium*, *Nitraria sphaerocarpa* Maxim., *Reaumuria songarica* (Pall). Maxim., *Salsola passerina* Bse. and other small shrubs and annual plants such as *Bassi adasyphylla* and *Eragrostis minor* Host. Icon. et Desert.

### Image pre-processing

Landsat 8 image was downloaded from the United States Geological Survey’s Earth Explorer website (https://earthexplorer.usgs.gov/) as lever 1 T processed scenes (track number 133-33), and the specific acquisition time is 28 August, 2015, cloud cover is less than 5.06% and distribute in southern part of the image far away from the study area. Before the identification of the oasis boundary, FLAASH atmospheric correction and image cropping of the study area were performed under ENVI 5.4. Then, using the object-oriented supervised classification to interpret the five types of ecological types (oasis, stony mountains, gravel desert, sandy desert and artificial sand fixation zone)^13^, and fusing the “noise” patches by dissolve command in ArcGIS. Because the basic unit of remote sensing data is a 30 × 30 m grid, it is impossible to form a smooth boundary between different ecosystems. Therefore, the positive and negative buffer analysis function is needed to build the smallest circumscribed polygon between the oasis and other ecosystems. Then the circumscribed polygon and the adjacent lines of each ecosystem represent the outer boundary of the oasis and which is also the inner margin of oasis-desert transition zone (red and yellow lines in Fig. 1). The main feature of the boundary is that the inner shelter forest, water area and desert inside oasis are mixed into the main body of oasis, and the shelter forest near the edge of oasis and the newly cultivated farmland near the main body of oasis are separated from the oasis.

### Calculation of NDVI and TCI

NDVI were calculated as follows:

1 $$NDVI=(Band5-Band4)/(Band5+Band4)$$

where Band5 and Band4 represent the red band and near-infrared band, respectively. According to Eq. (1), the NDVI of different types of transition zone were calculated.

The TCI were calculated using the following formula:

2 $$TCIs=(Band10-Ban{d}_{min})/(Band{10}_{max}-Band{10}_{min})$$

where TCIs represent the spatial temperature condition index, Band10 represent Landsat 8 data in band 10. Band10max and Band10min represent the maximum and minimum values of Band10, respectively.

3 $$TC{I}_{max}=a+bNDV{I}_{i}$$

4 $$TC{I}_{min}=a{\prime} +b{\prime} NDV{I}_{i}$$

In formula (3) and (4), TCI_max_ and TCI_min_ represent the maximum and minimum values of the temperature condition index, respectively when the data corresponding to the i^−th^ NDVI level in the study area is acquired. a, b, a′ and b′ are the coefficient to be determined. The NDVI classification of the oasis sandy desert transition zone and the oasis artificial sand-fixation transition zone is divided by 20 levels equidistantly. That is: i = 1, 2,…, 20.

TCI_max_ and TCI_min_ are two linear regression trend lines formed by the maximum and minimum values of different NDVI levels. The more significant the difference between the two trend lines, the greater the influence of surface non-vegetation information on TCI, and vice versa. Besides, if the NDVI value is larger than TCI_max_ or smaller than TCI_min_, it indicates that it has greater discrete (heterogeneous) at the corresponding NDVI level.

### Spatial buffer analysis

The use of medium-resolution remote sensing data to obtain vegetation information (species composition and community function) in the outer edge of the arid oasis is exceptionally different. It is not feasible to use these indicators to determine the gradient change in the transition zone. Therefore, we can only choose the relatively mature NDVI (representing vegetation productivity) to analyze the characteristics of vegetation productivity in the outer edge of the oasis^14^, because the low-resolution remote sensing data (MODIS-NDVI) can also obtain satisfactory results by studying vegetation productivity and ecological water demand^15^.

The buffer analysis of the oasis-desert transition zone was based on the oasis rim baseline, and two boundary types (red and yellow lines in Fig. 1) were selected for the oasis-natural sandy desert and oasis-artificial vegetation restoration areas. The sampling area is 2000 × 2500 m (Fig. 1). The analysis gradients are: in the range of 0–300 m, 30 m is the basic unit; 300–600 m, 60 m is the basic unit; 600–1000 m, 100 m is the basic unit; >1000 m, 200 m is the basic unit; the maximum buffer distance is 1600 m. Finally, the NDVI and TCI trends on the spatial gradient are obtained by buffer analysis to determine the width of transition zone.

### Scale effect

Focal analysis is one of the neighborhood analysis methods and is based on raster data analysis. The method is to use each grid as the analysis focus and calculate the statistical value of each grid within the defined range by the odd-number increase of the grid size (we selected the average value), which is an effective means to analyze the scale effect^16^. Besides, Landsat 8 has two thermal infrared bands, band 10 (wavelength range 10.6–11.2 μm) and band 11 (wavelength range 11.5–12.5 μm), which reflect surface temperature by brightness^5,8^. However, the band 11 is greatly affected by the atmospheric water vapor, then the band 10 can be used for the inversion of the surface temperature^5,11^. We use the minimum common divisor of approximate resolution between 90 m and 330 m as two scales to apply the focal analysis on NDVI and TCI, considering NDVI and TCI have different resolutions (with 30 m and 100 m respectively)^17,18^. Meanwhile, band 10 had already re-sampling in 30 m, and desert vegetation varied depending on scales. That is, NDVI and TCI in each grid represented by the average of all the grids within the range of 3 times and 11 times the grid side length of the original value, respectively.

All maps and figures were created using ArcGIS 10.6 (ESRI Inc., Redlands, California, USA) and MS Office Excel. All statistical analyses in this paper were conducted by SPSS 22 (IBM Inc., Armonk, New York, USA), and the significance check (F test) threshold was determined using P = 0.01 and P = 0.001. When P > 0.01, it is not significant; when 0.001 < P ≤ 0.01, it is significant; P ≤ 0.001 is very significant.

## Results

### Spatial pattern of NDVI and TCI in different transition zones

**Figure 2:**
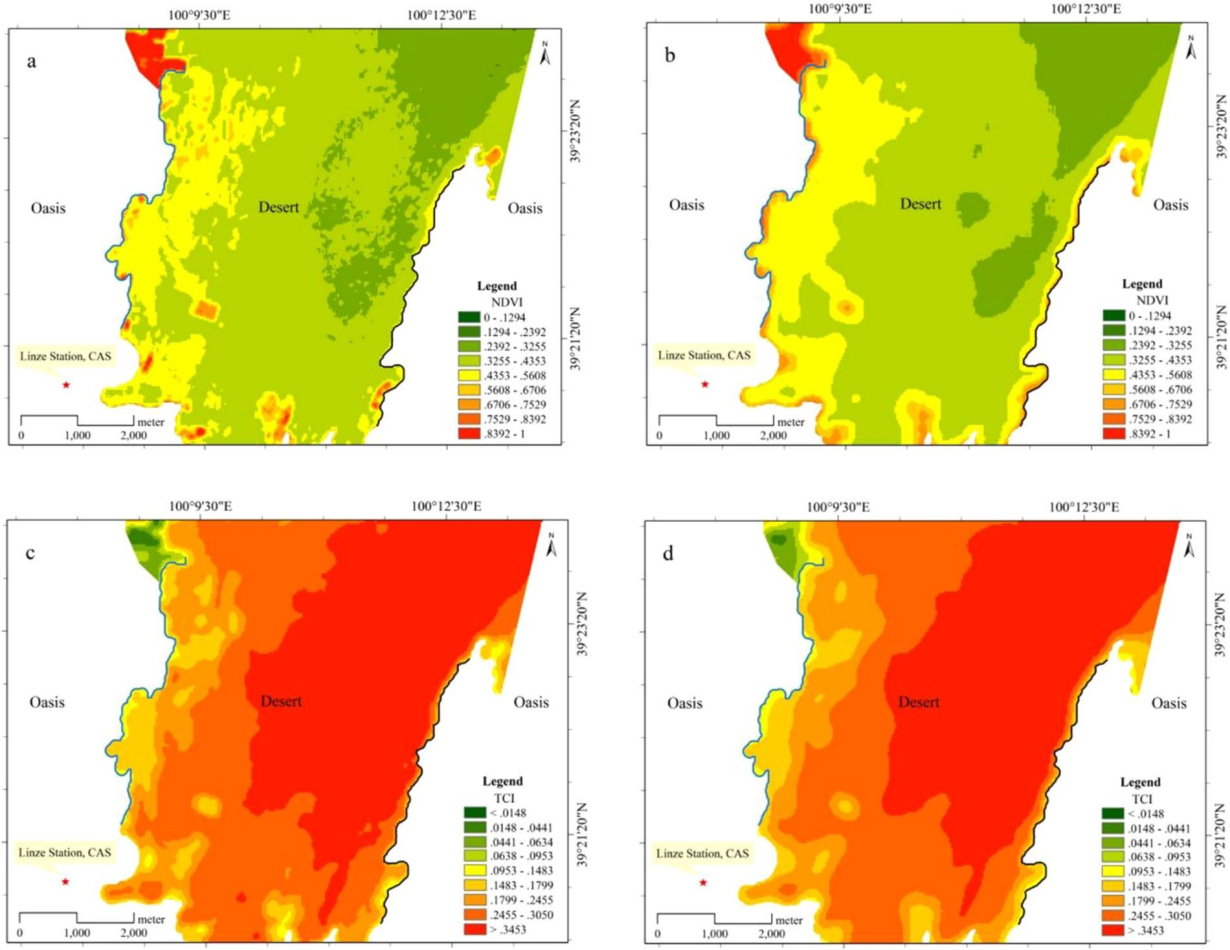
NDVI and TCI distribution maps of different scales in the oasis desert transition zone (**a**, NDVI 90 m; **b**, NDVI 330 m; **c**, TCI 90 m; **d**, TCI 330 m).

According to the results of NDVI and TCI analysis on different scales (Fig. 2), NDVI shows a decreasing trend from the oasis boundary to the desert, which trend is more obvious with the analysis scale from 90 m to 330 m (Fig. 2a,b). The trend of TCI, different from that of NDVI, increases from the edge of the oasis to the interior of the desert. This trend is also more prominent with increasing focal analysis (Fig. 2c,d). Comparing the two boundary types, the NDVI of the oasis-artificial sand-fixation transition zone (on the left side of Fig. 2) is much higher than the oasis-natural sandy desert transition zone (on the right side of Fig. 2) with NDVI from 0.0953 to 0.1483 occupying a large area. In contrast, TCI is significantly higher in the oasis-natural sandy desert transition zone than in the oasis-artificial sand-fixation transition zone with an absolute advantage of over 0.3453. In conclusion, NDVI and TCI are characterised by opposite trends on different scales in different transition zone types, laying a foundation for transition zone width identification.

**Figure 3:**
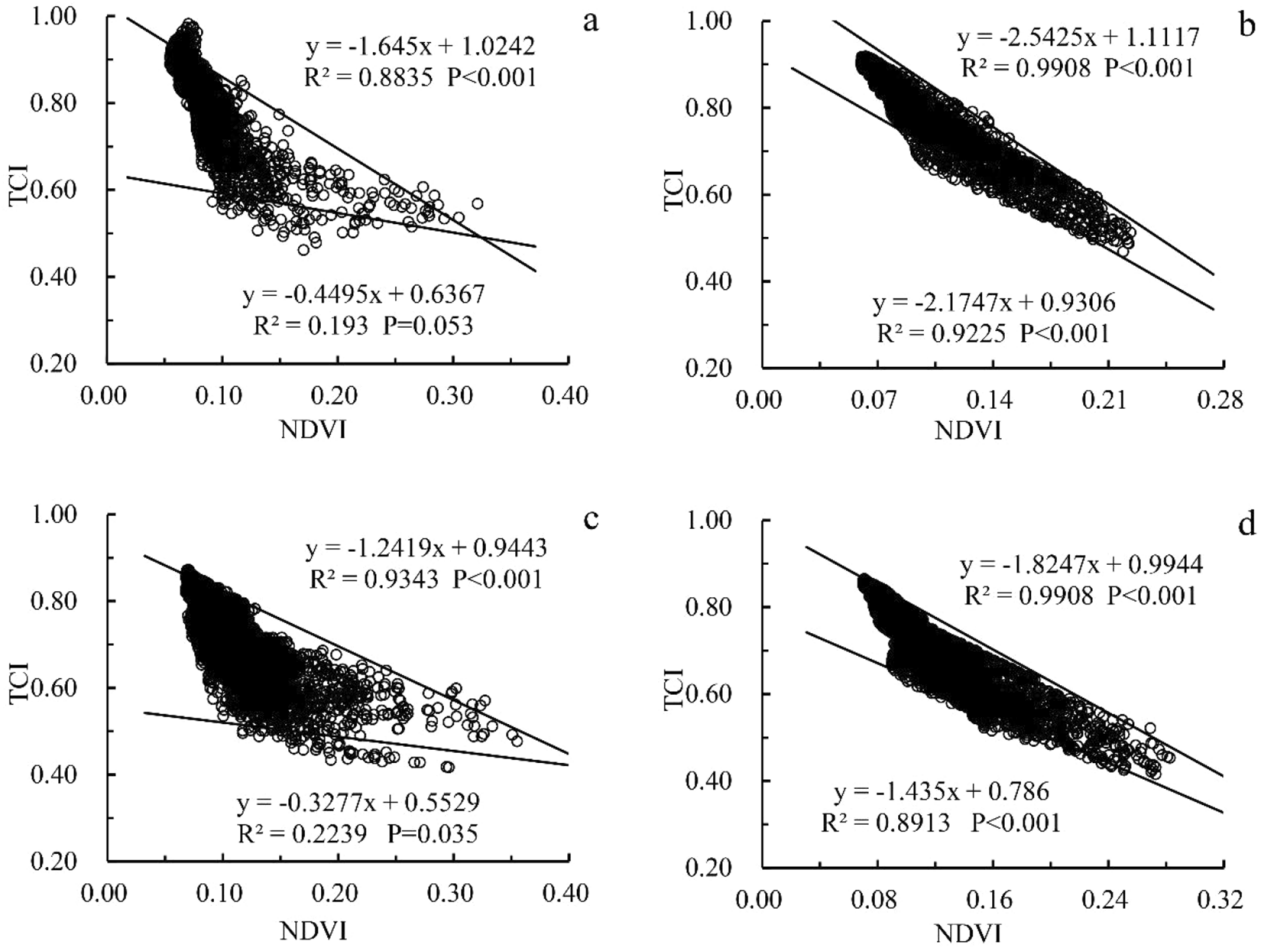
NDVI-TCI scatter plots of different resolutions in the oasis-sand desert transition zone (**a**, oasis sandy desert transition zone, resolution 90 m; **b**, oasis sandy desert transition zone, resolution 330 m; **c**, oasis sandy Desert artificial sand-fixation transition zone, resolution 90 m; **d**, oasis sandy desert artificial sand-fixation transition zone, resolution 330 m).

To validate the relationship between NDVI and TCI with different scales, we conducted a focused analysis to verify the differences between two types of transition zone. As shown in Fig. 3a, on the scale of 90 m, the TCI_max_ and TCI_min_ of oasis-sandy desert transition zone form a closed triangle which intersects at NDVI 0.32 and TCI 0.48. The slope of TCI_max_ is 3.66 times higher than that of TCI_min_, which means that the TCI_max_ increases with the NDVI in the transition zone with a greater the attenuation rate than that of the TCI_min_. In the oasis-artificial sand-fixation transition zone, TCI_max_ and TCI_min_ form an open triangle with no intersection between NDVI and TCI on the 90 m scale (Fig. 3c). The slope of TCI_max_ is 3.79 times higher than that of TCI_min_ with the TCI being the largest. The value increases with the NDVI and the decay rate is greater than the minimum. Also, seen from the TCI_max_ and TCI_min_ significance tests in Fig. 3a,c, the linear trend of NDVI and TCI_max_ is significant (P < 0.001) while the linear trend with TCI_min_ is not (P > 0.01). Thus, on the 90 m scale, the relationship between NDVI and TCI_min_ is relatively complex (not significant), and the relationship between NDVI and TCI_max_ is relatively simple (very significant). On the 330 m scale, TCI_max_ and TCI_min_ are approximately parallel within the study threshold, and the NDVI-TCI scatter is more convergent compared to the 90 m scale (Fig. 3b,d). The slope of TCI_max_ and TCI_min_ is 1.17 in the oasis-sand desert transition zone and 1.27 in the oasis-artificial sand fixation transition zone. With the increase of the analysis scale, the unsynchronization of the maximum and minimum attenuation of TCI with NDVI will significantly decrease. Moreover, it can be seen from Fig. 3b,d that the linear trend of NDVI with maximum and minimum TCI is significant (P < 0.001). Thus, on the 330 m scale, the relationship between NDVI and TCI_max_ and TCI_min_ is relatively simple and has a high degree of credibility.

### Width identification in different transition zones

**Figure 4:**
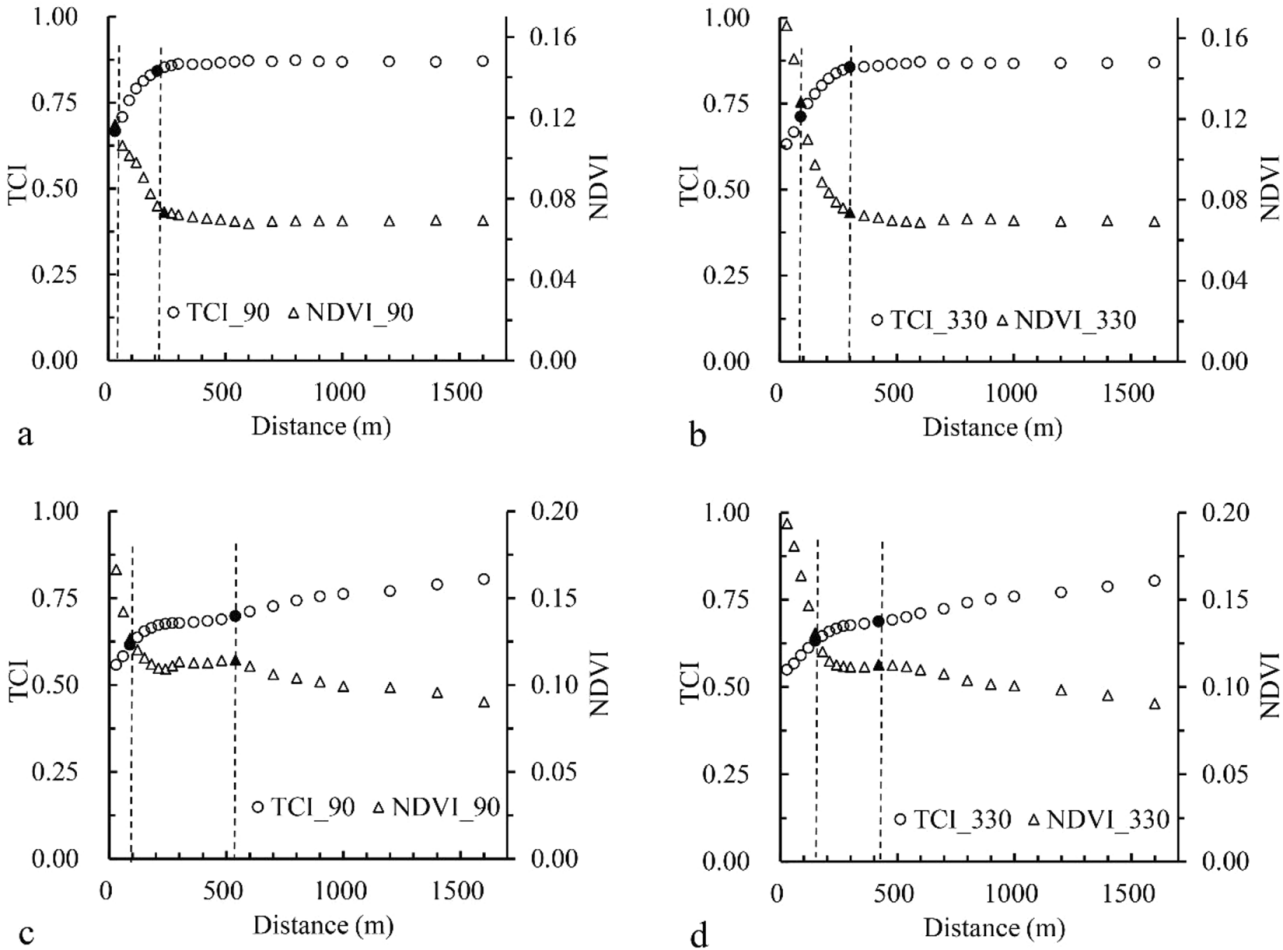
Spatial variation trend of NDVI-TCI with different resolutions in the oasis-sand desert transition zone (**a**, oasis sandy desert transition zone, resolution 90 m; **b**, oasis sandy desert transition zone, resolution 330 m; **c**, oasis sandy Desert artificial sand-fixation transition zone, resolution 90 m; **d**, oasis sandy desert artificial sand-fixation transition zone, resolution 330 m).

To calculate the width of different transition zones, the buffer analysis was applied to determine the intersections between NDVI and TCI that represent the width of the transition zones. In the oasis-sand desert transition zone, NDVI and TCI intersect at 30 m from the oasis boundary on the 90 m scale. TCI increases on the 30–220 m scale while NDVI shows a decreasing trend. When the buffer is over than 220 m, the two curves are substantially parallel (Fig. 4a). On the 330 m scale, the trend of the two curves resembles that of the 90 m scale with NDVI and TCI intersect at 90 m from the oasis boundary. However, the trend is opposite when NDVI and TCI intersect in the 90–300 m interval, but they also approximately parallel when the analysis scale is over 300 m (Fig. 4b).

In the oasis-artificial sand-fixation transition zone, the spatial variation trend of NDVI and TCI differs significantly from the sandy desert one. On the 90 m scale, the two intersect at 90 m from the oasis boundary with TCI and NDVI parallel in the 90–540 m interval. When the analysis scale is over 540 m, TCI shows an increasing trend while NDVI shows a decreasing trend (Fig. 4c). On the 330 m scale, the trend of the two curves resembles that on the 90 m scale. NDVI and TCI also intersect at 150 m with a shortened parallel interval. In the 150–420 m interval, the trend of TCI and NDVI are relatively parallel. When the buffer is over 420 m, the trend is consistent with that on the 90 m scale (Fig. 4d).

The analysis above shows that the spatial variation trend analysis of NDVI and TCI can determine the differences in spatial gradients of vegetation and meteorological indicators among diverse transition zone types based on the analysis scale. NDVI and TCI have a point of convergence in different transition zones, the two trends showed significant changes from the beginning to the intersection. The two indices also have an inflexion point, and the trend is inconsistent before and after the inflexion point (Fig. 4). In conclusion, the width of the oasis-sandy desert transition zone is significantly smaller than that of the artificial sand-fixation zone, and the trend is opposite to the increasing analysis scale.

## Discussion

The oasis-desert transition zone is significant in maintaining the stability of oasis and conserving ecosystem health. Recent years have seen studies in vegetation spatial heterogeneity, transitional vegetation productivity and landscape pattern change^3,5^. However, due to the lack of quantitative analysis of transition zone width in these studies, the sampling design could not be standardised within the threshold of the transition zone, weakening the comparability between research results. Thus, the methodological issue of standardising the width of the oasis-desert transition zone for research needs to be solved.

The remote sensing data analysis of vegetation temperature conditions has been widely used in the analysis of drought conditions in North America and water shortage in winter wheat production in China^5,19^. For example, Landsat 8, a satellite that can be used to analyse regional drought conditions, has an improved accuracy from NOAA-AVHRR 1.1 × 1.1 km to MODIS 1 × 1 km to 100 × 100 m. A series of research methods have also been developed, such as TCI, VTCI, and WSVI, using NDVI and ground brightness (indirect temperature or the brightness temperature converted by the Planck function) as the indicators^5,11,20,21^. The analysis of this study shows two opposite trend of NDVI and TCI (Fig. 2) with the 330 m scale more general than the 90 m scale and the NDVI and TCI gradients especially prominent in the oasis-desert transition zone. (Fig. 2b,d).

On the one hand, in the oasis-desert transition zone, although the TCI_max_ reaches a significant level, a strong theoretical constraint is on the maximum NDVI gradient. In fact, some NDVI are still between 0.06–0.10 and 0.28–0.32 above the TCI_max_ (Fig. 3a). However, this phenomenon disappeared on the 90 m scale of the oasis-artificial sand-fixation zone (Fig. 3c). On the other hand, it can be a useful constraint on the NDVI minimum, despite that the TCI_min_ relationship at the same scale is insignificant. The drawback is that NDVI values are smaller than TCI_min_ in the spectrum, and the NDVI in the sandy desert and artificial sand-fixation transition zone are at 0.09–0.23 and 0.12–0.30, respectively. The result is similar to the results of Patel (2012), Maduako (2017) and Khan (2018) in Gujarat, India, Enugu, Nigeria and Punjab Plain, Pakistan, where TCI_max_ and TCI_min_ have convergent constraints on NDVI-TCI in a foreseeable triangle^20,22–24^. However, the TCI_min_ correlation in this study is much lower than that of Patel’s (2012) LST(TCI)_min_, but its convergence is higher than other similar findings^24–26^ (they used a parallel X-axis that is smaller than the minimum TCI_min)_. This is due to the characteristic of the ecosystem in the oasis-desert transition zone where oasis and desert are very different in vegetation productivity and surface temperature, a difference that is the most significant in terrestrial ecosystems^4,17,18^, We can see from Fig. 3 that TCI decreases with the increase of NDVI, that is, the increase of surface vegetation cover will lead to the decrease of surface temperature.

Both TCI_max_ and TCI_min_ results are significant at the 330 m scale. It can be seen in Fig. 3b,d that TCI_max_ and TCI_min_ have a functional constraint on TCI and that TCI and NDVI are linearly distributed. This result indicates that when using large-scale analysis, the relationship between TCI and NDVI is less affected by spatial heterogeneity, which is conducive to identifying the overall trend. The result shows that the NDVI-TCI relationship reflected at the 330 m scale has integrated the discreteness of TCI_max_ and TCI_min_ on the 90 m scale, a consistent observation with the hierarchical structure theory’s small-scale variation irregularity which tends to increase the orderliness as the scale increases^18,27^. Besides, after comparing the two transition zones’ NDVI-TCI scatter and the distribution patterns of TCI_max_ and TCI_min_, it can be found that the oasis-sandy desert transition zone (Fig. 3a,b) is more discrete than the oasis-artificial sand-fixation transition zone (Fig. 3c,d) because in Fig. 3a,b, the points above TCI_max_ and below TCI_min_ are significantly more than in Fig. 3c,d, showing that the spatial heterogeneity of surface temperature in the oasis-sandy desert transition zone is more significant than that of the oasis-artificial sand-fixation area. This result is consistent with the variation of surface temperature in different ecosystems in Africa using MODIS data, meaning that the vegetation coverage is different from the ground temperature change in different ecosystems^26^. It should be emphasized that the relatively well soil moisture and vegetation conditions on the outer edge of the oasis may lead to an increase in surface evapotranspiration, increase the water content in the adjacent atmosphere and affect the accuracy of land surface temperature (brightness) retrieval^5,11^. However, this effect can be reduced to some extent by selecting bands that are less affected by moisture. At the same time, the difference of site conditions in this study shows that the discreteness of the distribution pattern of the oasis-sandy desert transition zone is more obvious than that of the oasis-artificial sand fixation area.

According to the results, on the 90 m analysis scale, the width of the oasis-sandy desert transition zone is 220 m and artificial sand-fixation zone 540 m; on the 330 m scale, the width of the oasis-sandy desert transition zone is 300 m and artificial sand-fixation zone 420 m. This phenomenon indicates that the scale effects are varied in different types of the transition zone. For the sandy desert transition zone, the width decreases as the analysis scale increases, while the trend of the oasis-artificial sand-fixation transition zone is the opposite. The width of the transition zone in the artificial sand fixation area at different scales is obviously larger than that in the sandy desert, which is directly related to the groundwater depth at the edge of the oasis, because the artificial sand fixation in the arid area is chosen in the area with shallow groundwater table. Groundwater supply is the most important factor to ensure the control effect of artificial vegetation. The changes of NDVI and TCI have two characteristics. First, NDVI and TCI have a steep intersection, indicating a rapid change of vegetation and surface temperature near the edge of the oasis in the transition zone; second, an inflexion point appears after the intersection. The comparison of the two transition zones show that under different analysis scales, the steep transition point of the sandy desert transition zone is closer to the edge of the oasis, they are 30 m and 90 m, respectively (Fig. 4a,b); while the transition zones of the artificial sand-fixation zone are far apart, they are 90 m and 150 m, respectively (Fig. 4c,d). It is noticeable that NDVI and TCI trends in two transition zone types are different with the changes of two indicators in the transition zone of sandy deserts tending to be parallel as the distance from the oasis boundary increases. However, the trend in the oasis-artificial sand-fixation area is less salient, indicating an increasing survival rate and preservation rate of artificial vegetation adjacent to the sandy desert. The transition zone of desert oasis has important practical significance for maintaining the stability of ecological environment. The identification of the width of the transition zone for two different ecological regions plays an important guiding role in maintaining the stability and biodiversity of the local ecosystem. At the same time, in the arid inland areas, water source is often an important factor that determines the growth of plants, surface precipitation and groundwater affect the growth of vegetation at the same time, and the height of groundwater in the oasis-desert transition zone is different from that in the oasis-artificial sand fixation area, which also directly leads to the different spatial distribution pattern of aboveground vegetation, which has an impact on the distribution location of the transition zone.

Our study made an improvement for width identification of different transition zones, and the results of different width among two types transition zone could provide references for oasis protection. This paper considers dry edge factors, but the accuracy of the width is still affected by climate factors. In the further studies we will take consideration of precipitation so that it can further improve the accuracy of detecting the width.

At the same time, taking the desert-oasis transition zone as an example, the clear spatial definition of the ecological ecotone of different ecosystems can provide some ideas for other similar environments, such as forest-grass junction zone, forest-field junction zone, snow line monitoring and so on. it is of great significance to maintain the stability of the ecological environment and control the impact of human activities on the natural environment.

## Conclusions

In the oasis-desert transition zone, NDVI and TCI can capture the different trends of vegetation and surface temperature in spatial gradients. TCI_max_ and TCI_min_ analyses indicate that TCI_max_ and TCI_min_ can constrain this range of trend variation, and the effect increases with the analysis scale. On the same spatial scale, NDVI and TCI have opposite trends and have intersections. Among them, the intersection of the sandy desert transition zone is between 30–90 m, and the oasis artificial sand-fixation zone is between 90–150 m. The width of different transition zone types is different and affected by scale effect. The width of the sandy desert transition zone is between 220–300 m, and the width increases with the increase of analysis scale. The oasis artificial sand-fixation zone is between 420 and 540 m, which decreases with the increase of the analysis scale.

## Data Availability

The Landsat data are publicly available here: https://earthexplorer.usgs.gov/.

## References

[1] Li, X., Yang, K. & Zhou, Y. Progress in the study of oasis-desert interactions (2016).

[2] Mao D (2014). Characteristics of wind erosion and deposition in oasis-desert ecotone in southern margin of Tarim Basin, China. Chinese geographical science.

[3] Chen J, Hu Y, Lü S, Yu Y (2014). Influence of advection on the characteristics of turbulence over uneven surface in the oasis and the Gobi Desert. Science China Earth Sciences.

[4] Matchanov M, Teodoro A, Schroder C (2016). Criterion definition for the identification of physical-geographical boundaries of Khorezm oasis through remotely sensed data. Environmental monitoring and assessment.

[5] Kogan FN (1995). Droughts of the late 1980s in the United States as derived from NOAA polar-orbiting satellite data. Bulletin of the American Meteorological Society.

[6] AghaKouchak A (2015). Remote sensing of drought: Progress, challenges and opportunities. Reviews of Geophysics.

[7] Arekhi, M., Saglam, S. & Ozkan, U. Y. Drought monitoring and assessment using Landsat TM/OLI data in the agricultural lands of Bandar-e-Turkmen and Gomishan cities, Iran. *Environment*, *Development and Sustainability*, 1–18 (2019).

[8] Wan Z, Wang P, Li X (2004). Using MODIS land surface temperature and normalized difference vegetation index products for monitoring drought in the southern Great Plains, USA. International journal of remote sensing.

[9] Bandos TV, Bruzzone L, Camps-Valls G (2009). Classification of hyperspectral images with regularized linear discriminant analysis. IEEE Transactions on Geoscience and Remote Sensing.

[10] Carlson TN, Gillies RR, Perry EM (1994). A method to make use of thermal infrared temperature and NDVI measurements to infer surface soil water content and fractional vegetation cover. Remote sensing reviews.

[11] Jain SK, Keshri R, Goswami A, Sarkar A (2010). Application of meteorological and vegetation indices for evaluation of drought impact: a case study for Rajasthan, India. Natural hazards.

[12] Zhao W, Chang X (2014). The effect of hydrologic process changes on NDVI in the desert-oasis ecotone of the Hexi Corridor. Science China Earth Sciences.

[13] Wang, J. 1:100,000 scale desert distribution mapset of China, http://westdc.westgis.ac.cn (2006).

[14] Li, F., Zhao, W., & Liu, H. The response of aboveground net primary productivity of desert vegetation to rainfall pulse in the temperate desert region of northwest China. *PLoS One*, **8** (2013). 10.1371/journal.pone.0073003 PMC3760839 24019888

[15] Zhao W (2018). Estimating water consumption based on meta-analysis and MODIS data for an oasis region in northwestern China. Agricultural Water Management.

[16] Gökgöz T, Baker MKM (2015). Large scale landform mapping using Lidar DEM. ISPRS International Journal of Geo-Information.

[17] He Z, Zhao W, Chang X, Chang X, Fang J (2006). Scale dependence in desert plant diversity. Biodiversity & Conservation.

[18] Levin SA (1992). The problem of pattern and scale in ecology: the Robert H. MacArthur award lecture. Ecology.

[19] Liu X (2016). Agricultural drought monitoring: Progress, challenges, and prospects. Journal of Geographical Sciences.

[20] Patel NR, Parida BR, Venus V, Saha SK, Dadhwal VK (2012). Analysis of agricultural drought using vegetation temperature condition index (VTCI) from Terra/MODIS satellite data. Environmental monitoring and assessment.

[21] Gidey E, Dikinya O, Sebego R, Segosebe E, Zenebe A (2018). Analysis of the long-term agricultural drought onset, cessation, duration, frequency, severity and spatial extent using vegetation health index (VHI) in Raya and its environs, northern Ethiopia. Environmental Systems Research.

[22] Khan J, Wang P, Xie Y, Wang L, Li L (2018). Mapping MODIS LST NDVI imagery for drought monitoring in Punjab Pakistan. IEEE Access.

[23] Maduako IN, Ndukwu RI, Ifeanyichukwu C, Igbokwe O (2017). Multi-index soil moisture estimation from satellite earth observations: comparative evaluation of the topographic wetness index (TWI), the temperature vegetation dryness index (TVDI) and the improved TVDI (iTVDI). Journal of the Indian Society of Remote Sensing.

[24] Wang L (2019). Monitoring maize growth conditions by training a BP neural network with remotely sensed vegetation temperature condition index and leaf area index. Computers and Electronics in Agriculture.

[25] Sun W (2008). Using the vegetation temperature condition index for time series drought occurrence monitoring in the Guanzhong Plain, PR China. International Journal of Remote Sensing.

[26] Vancutsem C, Ceccato P, Dinku T, Connor SJ (2010). Evaluation of MODIS land surface temperature data to estimate air temperature in different ecosystems over Africa. Remote Sensing of Environment.

[27] Wu, J. & Hobbs, R. J. (Eds.). Key Topics In Landscape Ecology. Cambridge University Press. (2007).

